# Emotional Intelligence Profiles in College Students and Their Fathers’ and Mothers’ Parenting Practices

**DOI:** 10.1007/s10804-018-9286-0

**Published:** 2018-01-29

**Authors:** Maria Cruz García Linares, Maria Villa Carpio Fernández, Maria Teresa Cerezo Rusillo, Pedro Félix Casanova Arias

**Affiliations:** Dpto. de Psicología, Universidad de Jaén, edificio C5, Paraje Las Lagunillas s/n, 23071 Jaén, Spain

**Keywords:** Adolescence, Parenting practices, Emotional intelligence

## Abstract

The present study has two objectives: first, to analyze whether the dimensions that make up emotional intelligence (attention, clarity, and repair) give rise to different profiles of university students, and secondly, to determine whether these different profiles are differentially associated with the parenting practices that students report with regard to their fathers and mothers. Results obtained indicate the existence of different profiles of college students. The profile that corresponds to adequate emotional skills presents a lower score in attention, but higher scores in clarity, and especially in mood repair. The other two profiles are inadequate, in the first case because a higher score in emotional attention is accompanied by low scores in mood repair, and in the second case because low scores are presented in all three dimensions. Likewise, we verified the existence of significant differences in the educational practices of parents, the adequate profile is characterized by greater use of parenting dimensions considered to be positive, and at the same time, lower scores on dimensions considered to be negative. One of the dysfunctional profiles is associated with higher scores in positive practices, and is also associated with higher scores in practices considered to produce a negative effect. The second dysfunctional profile is associated with higher scores on the dimensions considered to be negative and lower scores on positive dimensions.

## Introduction

As Manzeske and Stright (2009) indicate, one important aspect of development during early adulthood is the use of skills to regulate both positive and negative emotions in social, educational, and professional contexts.

This set of skills has received the name emotional intelligence, and has become one of the top fields attracting scientific interest in recent decades. According to the Mayer and Salovey model (1997), the main dimensions of emotional intelligence are perceiving, understanding, and regulating emotions. *Perceiving* refers to the ability to precisely perceive and name emotional expressions and situational and behavioral signs. *Understanding* refers to being able to identify one’s own and others’ emotions, and *regulating* emotions is the ability to cope with negative emotions and encourage positive ones.

However, as Alegre and Benson (2010) indicate, the three dimensions of emotional intelligence seem to be differently related to psychological adjustment. While the dimensions of understanding and mood repair (regulating emotions) generally predict the degree of psychosocial adjustment (Fernandez-Berrocal and Extremera 2007; Wong et al. 2007; Berking et al. 2008), the relationship between the attention (perceiving) dimension and adjustment problems is not clear; contradictory results show that attention to feelings may play a different role than understanding and mood repair. Therefore, we concur with Alegre and Benson (2010) that the components of emotional intelligence must be studied separately.

Among the many factors that may determine the emotional development of children and youth, the role of the family must be emphasized. Indeed, the influence of the family context in children’s emotional development has been pointed out by diverse authors, whether that influence comes from what parents teach, or from the parents’ own behavior. For example, Mayer and Salovey (1997) indicate that interactions within the family context mark the beginning of emotional skill acquisition, with parents teaching their children to connect their emotions with social situations that they experience.

One of the aspects of parents’ behavior that has great influence on children’s development is parenting style (Maccoby and Martin 1983), for which two important dimensions have been identified—affect and control (Grolnick and Gurland 2002).

The first of these dimensions refers to the parents’ availability and their demonstration of support and affection for their children. This set of positive practices has been related to a lower rate of externalizing problems in children, to higher self-esteem, and to good psychological adjustment (Eiden et al. 2007; Khaleque et al. 2007; Rohner 1990).

The second dimension concerning parenting styles is control, which is a more complex construct. According to Alegre (2011), some practices that make up this construct show positive relationships to developmental outcomes, while other practices show negative relationships or undesirable outcomes. The former would include behavior control and support for autonomy, along with inductive discipline (De Clercq et al. 2008; Sanders 2008). By contrast, practices that produce negative outcomes include most notably psychological control and harsh discipline (Barnett et al. 2008; Barry et al. 2007; Shelton and Harold 2008).

Despite extensive research on how parenting styles influence many aspects of the development and psychological adjustment of children and youth, little attention has been given to the relationship and influence of parenting styles on the development of emotional intelligence (Alegre 2011). Furthermore, most of the research has focused particularly on children and adolescents, with less attention given to these relationships in youths who are entering early adulthood (Morris et al. 2007).

According to Alegre and Benson (2010), there is evidence that the two main dimensions of parenting styles mentioned above are related to children’s emotional intelligence. Namely, parental affect has proven to be positively related to children perceiving, understanding, and regulating emotions (Alegre and Benson 2007; Bennett et al. 2005; Eisenberg et al. 1998; Steele et al. 1999). There are also data that show parental control to be related to children perceiving, understanding, and regulating emotions (Morris et al. 2007; Pears and Moses 2003; Perlman et al. 2008).

However, as indicated above, the construct of parental control is quite complex and includes a number of practices with different outcomes that need to be analyzed.

Manzeske and Stright (2009) indicate that in early adulthood there are two important types of control: behavior control and psychological control (Barber et al. 1994). On one hand, moderate levels of behavior control have been associated with positive emotional adjustment in children (Barber et al. 2005). On the other hand, psychological control is a parental behavior that interferes with the adolescent’s emotional development, restricting emotional intelligence and diminishing the competency to establish and express one’s own feelings. Psychological control interrupts the development of emotional autonomy by interfering with adolescents’ ability to establish and express their thoughts and feelings (Barber and Harmon 2002; Manzeske and Stright 2009; Moilanen 2007).

Despite the demonstrated negative influence of parents’ psychological control on adolescents’ emotional development (Barber and Harmon 2002), few studies have investigated its impact on their emotional intelligence. In the study by Gugliandolo, Costa, Cuzzocrea, and Larcan (2015), parents’ psychological control was shown to be negatively associated with the children’s emotional intelligence.

More recently, authors like Kerr and Stattin (2000) have proposed a new concept concerning parental control, relating to how well parents know their children; it involves the parents’ asking for information and the children revealing it. This form of control can be especially important when children are older.

Similarly, beginning in adolescence, autonomy support through respect for children’s independence and decision making constitutes one component of an adequate parenting style (Steinberg et al. 1992).

With respect to parental discipline, punitive and harsh discipline has been shown to be related to low levels of emotional understanding and regulation in children (Morris et al. 2007; Pears and Moses 2003).

However, there are diverse parenting practices whose influence on emotional intelligence has not been investigated. Namely, there are few or no studies on how behavior control, psychological control, autonomy support, or revelation relate to emotional intelligence.

Another important aspect has to do with whether it is useful to differentiate between fathers’ and mothers’ parenting styles; some results have shown that the two parents’ practices may differently influence their children (García-Linares et al. 2011; Lim et al. 2015; Milevsky et al. 2007).

Considering all the above, a number of important questions follow in regard to the scarcity of research on how certain parenting practices relate to their children’s emotional intelligence, especially in early adulthood; on the differential behavior of the three main dimensions of emotional intelligence in relation to psychological adjustment; and on the differing influence of fathers’ and mothers’ parenting styles.

These questions constitute the basis for carrying out the present study, which analyze relations between a substantial number of fathers’ and mothers’ parenting practices (affect, inductive discipline, strict discipline, permissive discipline, autonomy support, behavior control, psychological control, and revelation) and the main dimensions of emotional intelligence, in college students. Specifically, the objectives of this study are as follows:

One: To verify whether different types of profiles emerge when combining the dimensions that make up emotional intelligence (perception, clarity, and repair) in college students.

Two: To determine whether there are differences between the established groups, in fathers’ and mothers’ parenting practices, as reported by the students.

## Method

### Participants

Participating in the study were 300 freshmen students from different degree programs, all of them within the School of Humanities and Educational Sciences at the University of Jaen. Of the total sample, 5.7% of students were enrolled in the Art History degree, 11% in English Studies, 38% in Psychology, 24% in Early Childhood Education, and 21.3% in Primary Education. The gender distribution was 24.3% male and 75.7% female.

Mean age of the sample was 19.9 years, with a standard deviation de 1.31. The age distribution was 38% 18-year-olds, 25% 19-year-olds, 14.3% age 20, 7% age 21, and 5% age 22; the remaining 10.7% were age 23 or older.

Regarding their father’s educational level, 32.7% had a high school education, 22.3% primary school education, 20.7% had vocational training, 11.35% had a bachelor’s degree, 9% had not completed any level of education, and 4% had postgraduate studies. Regarding the mother’s educational level, 34.7% had a high school education, 23.7% primary school education, 22% had vocational training, 13.3% had a bachelor’s degree, 6% had not completed any level of education, and 0.3% had postgraduate studies.

Incidental sampling was used to form the study sample; the degree programs and groups were selected according to their availability. Participants were asked for their written consent. The questionnaires were completed anonymously.

The tests were applied during a single hour-long session, held during class hours within the different degree programs (Art History, English Studies, Early Childhood Education, and Primary Education), with both the professor and the researchers present. Students’ collaboration was solicited and they were assured that questionnaire responses were entirely voluntary and confidential.

### Instruments

In order to assess the different forms of discipline, we used the *Escala de normas y exigencias* (hereafter, Rules and Demands scale) (Bersabé et al. 2001; Fuentes et al. 1999), which contains three factors or dimensions: inductive discipline (e.g., “He/she reasons with me and we agree on the rules”), strict discipline (e.g., “He/she requires me to follow rules even if I don’t understand them”), and permissive discipline (e.g., “He/she doesn’t care whether I obey or disobey”). The first two factors each have ten items, while the third factor has eight. All the dimensions are scored on a five-point Likert scale (from 1 = “never” to 5 = “always”). The first two scales produce a total score between 10 and 50, while the total score for the permissive discipline scale falls between 8 and 40. A higher score corresponds to a higher degree of the aspect being assessed. Assessment of parenting practices is done separately for the father and the mother. Internal consistency indices (Cronbach alpha statistic) for the dimensions of inductive, strict, and permissive discipline, relatively, were as follows: for mothers, .85, .74, and .72; and for fathers, .86, .74, and .73. The instrument’s convergent validity was obtained using correlation analysis of the scores in the discipline dimensions of the Rules and Demands scale and the factors assessed with the Parental Authority Questionnaire (Buri 1991). Values ranging from 0.38 for permissive discipline and 0.63 for inductive discipline were obtained (Bersabé et al. 2001). The utility of these scales has been proven in several studies that analyze the influence of parenting practices on the functioning of adolescents (De la Torre et al. 2011; García-Linares et al. 2014).

In order to assess the remaining parenting practices, we used the *Escala para la evaluación del estilo parental* (Oliva et al. 2007), or “Parenting Style Assessment Scale.” This scale evaluates separately the father’s and mother’s affect and different types of control. It contains 41 items where the adolescent assesses six dimensions of mother’s and father’s style on an independent basis. Internal consistency of dimensions was high: Affect and communication (*α* = 0.92), Promotion of autonomy (*α* = 0.88), behavior control (*α* = 0.82), psychological control (*α* = 0.86), revelation (*α* = 0.85), and Humor (*α* = 0.88). For the present study, we used only the following dimensions: Affect and communication, Promotion of autonomy, Behavior control, Psychological control, and Revelation, which together completed the construct control, along with the three forms of discipline assessed by the instrument described above.

The Trait Meta-Mood Scale-24 (TMMS 24; adaptation by Fernández-Berrocal et al. 2004) was used to assess emotional intelligence. The questionnaire is composed of 24 items. Participants are asked to evaluate the degree to which they agree with each of the items on a five-point Likert scale, ranging from *Totally disagree* (1) to *Totally agree* (5). The scale comprises three subfactors: Attention to one’s own feelings, emotional clarity, and mood repair. Fernández-Berrocal et al. (2004) found internal consistencies of .90 for Attention, .90 for Clarity, and .86 for Repair.

### Statistical Analyses

Due to the different behaviors of the Emotional Intelligence dimensions of attention, clarity, and repair, we then used the cluster analysis technique in order to determine whether different profiles existed in the grouping of these dimensions, as has been done in prior research (García-Férnandez et al. 2015; García-Linares et al. 2015; Gázquez et al. 2015).

The criteria used were the scores obtained on the three dimensions of emotional intelligence assessed by the TMMS-24, that is, attention to emotions, emotional clarity, and repair. Maximizing intergroup differences was the criterion for determining the number of clusters. In addition to this criterion, we also took into account theoretical viability and psychological significance of each of the groups represented by the different profiles of emotional intelligence.

After establishing the different groups by cluster analysis, analyses of variance (ANOVAs) were carried out in order to analyze the statistical significance of between-group differences in parenting practices. In order to analyze the magnitude or effect size of these differences, we considered the *η*^2^ index.

After examining the analyses where there were statistically significant differences, post hoc tests were performed to identify which groups were differentiated by these differences. Data were analyzed using the Statistical Package for the Social Sciences, version 20.0 (IBM, 2011).

## Results

**Chart 1:**
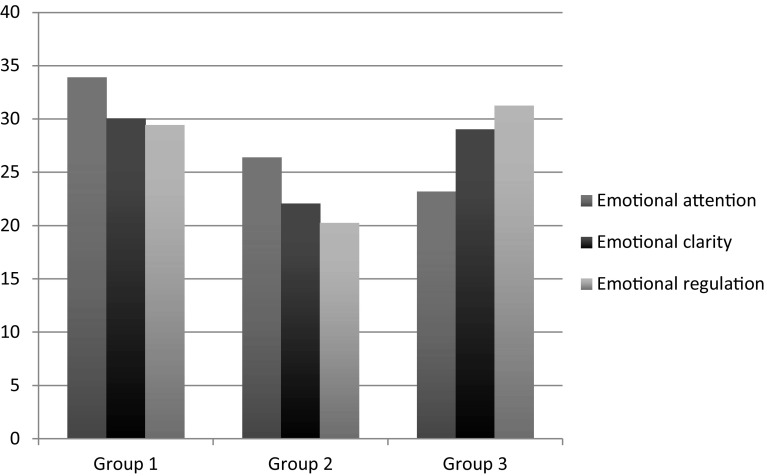
Emotional intelligence profiles obtained. Group 1: greater attention and emotional clarity, lower in mood repair. Group 2: low attention, clarity, and mood repair. Group 3: lowest in attention to emotions and highest in repair

Having completed the cluster analysis, shown in Chart 1, three profiles (or groups) were obtained. Upon comparison, the first group has the highest score in the attention and clarity dimensions, and a lower score for repair. The second group presents lower scores in attention, clarity, and repair. Finally, the third cluster has the lowest score in attention, and most especially, the highest score in repair.

Once the clusters were identified according to scores on the emotional intelligence dimensions, analyses of variance (ANOVAs) were carried out to analyze the between-group differences produced in the dependent measures of father’s and mother’s parenting practices.

Regarding the mother’s parenting practices, results indicate that there are significant differences in the dimensions of inductive discipline F(2,297) = 8.2, *α* = 0.000; affect F(2,297) = 5.45, *α* = 0.005; and autonomy support F(2,297) = 4.9, *α* = 0.008.

Regarding the father’s parenting practices, results indicate that there are significant differences in the dimensions of inductive discipline F(2,297) = 13.8, *α* = 0.000; strict discipline F(2,297) = 5.09, *α* = 0.007; affect F(2,297) = 9.8, *α* = 0.000; behavior control F(2,297) = 3.8, *α* = 0.024; autonomy support F(2,297) = 13.4, *α* = 0.000; psychological control F(2,297) = 4.8, *α* = 0.009; and revelation F(2,297) = 3.8, *α* = 0.023.

With regard to inductive discipline in both father and mother, a posteriori comparisons using the DSM statistic indicate that Cluster 2 has a lower score than Clusters 1 and 3. Regarding strict discipline from the father, the Cluster 3 score is significantly lower than scores for Clusters 1 and 2 (see Table 1).

**Table 1:** Means, standard deviations, contrast value, statistical significance, and post hoc comparisons in parenting practices as a function of the different groups

	Gr 1	Gr 2	Gr 3	*F*	*p*	*post hoc* comparisons	*η* ^2^
	*M* (*SD*)	*M* (*SD*)	*M* (*SD*)				
Mother’s parenting							
Affect	34.39 (6.6)	31.68 (6.6)	34.41 (5.9)	5.45	.005	G2 < G1 (.005) G2 < G3 (.003)	.035
Behavioral control	21.01 (4.8)	20.53 (5.04)	19.82 (5.33)	1.48	n.s		
Promotion of autonomy	32.77 (6.5)	29.77 (6.75)	31.85 (6.43)	4.9	.008	G2 < G1 (.002) G2 < G3 (.026)	.032
Psychological control	19.78 (5.5)	20.01 (5.48)	18.63 (5.12)	2.014	n.s		
Revelation	18.71 (5.26)	17.63 (4.62)	18.79 (4.7)	1.487	n.s		
Inductive discipline	39.55 (7.66)	35.36 (7.55)	39.10 (7.54)	8.2	.0000	G2 < G1 (.000) G2 < G3 (.001)	.052
Strict discipline	25.29 (7.25)	25.27 (7.16)	23.73 (6.68)	1.77	n.s		
Permissive discipline	18.17 (5.09)	17.54 (4.46)	18.34 (4.90)	.700	n.s		
Father’s parenting							
Affect	33.03 (6.17)	28.64 (7.66)	32.35 (7.37)	9.83	.000	G2 < G1 (.000) G2 < G3 (.000)	.062
Promotion of autonomy	32.54 (5.79)	27.59 (7.31)	31.33 (6.79)	13.4	.000	G2 < G1 (.000) G2 < G3 (.000)	.073
Behavioral control	19.70 (5.19)	18.93 (5.80)	17.66 (5.57)	3.8	.024	G1 > G3 (.007)	.023
Psychological control	19.12 (5.55)	19.97 (5.68)	17.66 (5.04)	4.8	.009	G3 < G1 (.049) G3 < G2 (.003)	.030
Revelation	17.22 (5.01)	15.28 (5.43)	17.04 (5.08)	3.82	.023	G2 < G1 (.013) G2 < G3 (.018)	.018
Inductive discipline	39.32 (7.76)	33.10 (8.08)	37.59 (8.44)	13.8	.000	G2 < G1 (.000) G2 < G3 (.000)	.085
Strict discipline	24.20 (7.53)	25.34 (7.35)	22.25 (6.31)	5.09	.007	G3 < G1 (.042) G3 < G2 (.002)	.033
Permissive discipline	18.15 (4.91)	17.38 (4.58)	18.27 (5.13)	.877	n.s		

Group 1: greater attention and emotional clarity, lower in emotional regulation. Group 2: low attention, clarity, and emotional regulation. Group 3: lower attention to emotions and greater mood repair

*p < 0.05; **p < 0.01; ***p < 0.001

Regarding strict discipline from the father, Cluster 2’s score is significantly lower than scores for Clusters 1 and 3. In the autonomy support dimension, for both father and mother, Cluster 2’s score is lower than this same score in Clusters 1 and 3.

In father’s behavior control, the score for Cluster 1 is higher than for Cluster 3; in father’s psychological control, the score for Cluster 3 is lower than for Clusters 1 and 2. Finally, in the dimension of father revelation, Cluster 2 has a lower score than Clusters 1 and 3.

## Comments

Regarding our first objective, the results of this study show that we obtain different profiles or groupings by combining the three dimensions that comprise emotional intelligence: attention, clarity, and repair. The three-cluster solution that was selected gives us one pattern that we might describe as dysfunctional, in that it presents higher scores for emotional attention, but lower scores for repair. The second pattern obtained is characterized by low scores on all three dimensions, that is, we might interpret this profile as persons who have few emotional skills in general. Finally, the third profile obtained is characterized by a lower score in attention but higher scores in clarity, and especially in mood repair, thus corresponding to a profile of adequate emotional skills.

The interpretation of these profiles is based on data from several investigations where people with high emotional intelligence present a profile with moderate-to-low scores in emotional attention and high scores in understanding and regulating emotions; such a profile is associated with better adjustment and psychosocial well-being (Extremera and Fernández-Berrocal 2002; Salguero et al. 2012).

For example, the dimensions of clarity and mood repair were found to be related to life satisfaction measures, that is, persons who present high levels of clarity and repair manifest greater life satisfaction (Augusto et al. 2006; Cerezo et al. 2016; Extremera et al. 2005; Páez et al. 2006; Palomera and Brackett 2007).

With regard to attention given to the emotions themselves, several research studies report that people who present high levels of attention to emotions manifest a greater number of physical symptoms, depression, anxiety, and a deficit in their physical and social functioning (Extremera and Fernández-Berrocal 2002; Goldman et al. 1996; Salovey et al. 2002; Thayer et al. 2003).

Regarding our second objective, our analysis of fathers’ and mothers’ parenting practices reveals significant differences between profiles, and so validates their existence. Specifically, the first profile is associated with higher scores in practices considered to be positive, such as inductive discipline, affect, and autonomy support from father and mother, together with revelation and behavior control on the father’s part. However, this profile was described as dysfunctional, since it is also associated with higher scores in practices considered to produce a negative effect on psychological development, such as father’s strict discipline and psychological control.

The second profile is associated with lower scores on dimensions considered positive: inductive discipline, affect, and autonomy support from father and mother, and revelation on the father’s part. Moreover, this profile is associated with higher scores on the dimensions considered to be negative, that is, father’s strict discipline and psychological control.

Finally, the third profile or cluster, corresponding to an adequate combination of emotional skills, is associated with higher scores on dimensions considered positive: inductive discipline, affect, and autonomy support from father and mother, and at the same time, lower scores on the dimensions considered to be negative, namely, father’s strict discipline and psychological control.

Therefore, the results indicate that an adequate combination of parenting practices, with more use of positive practices and less use of negative practices, is associated with a profile of adequate emotional skills in one’s offspring. An inadequate combination of parenting practices, with more use of negative practices and less use of positive practices, is associated with a profile characterized by deficient emotional skills. Finally, what we might refer to as an inconsistent use of parenting practices, given that both positive and negative practices are used, is associated with what we consider to be a dysfunctional profile, characterized by a higher level of emotional attention but lower mood repair skills.

Although there are no similar research studies with which to compare our results, there are studies that have examined the differences between emotional intelligence profiles with respect to other variables. For example, the study from Gázquez et al. (2015) analyzes adolescents’ social behavior and confirms the existence of the three profiles that we found, as well as a fourth profile characterized by high skills in all three dimensions of EI. Students whose profiles correspond to what we call the dysfunctional profile (higher attention and lower repair) and the inadequate profile (low attention, clarity, and repair) show more social inhibition than the remaining students, and the dysfunctional profile also presents a higher level of social anxiety.

Similarly, the study from García-Fernández et al. (2015) analyzes adolescents’ learning strategies, and confirms the existence of the same three profiles, as well as a fourth profile characterized by high scores on all three dimensions of EI. This study confirms that students with the adequate profile (low attention and greater repair) use learning strategies to a greater extent than do students with the dysfunctional or inadequate profile. Their results indicate that low levels of attention accompanied by high levels of mood repair improve learning processes, while high levels of attention with low levels of repair hinder adolescents’ social and academic adjustment.

Therefore, the studies mentioned ratify the existence of the same profiles found in our study, and confirm that the profile showing lower attention and greater repair is associated with positive results, while the profile with greater attention and lower repair is associated with negative results.

Our study differs from these other studies in that we did not find the profile characterized by high scores in all three dimensions of emotional intelligence. This result may perhaps be explained by the difference in sample: in our case the participants were university students, while the two studies above assessed secondary students.

The distinction between father’s and mother’s parenting practices made it possible to obtain results that point toward greater relevance of the father’s role in comparison to the mother’s. With regard to the father’s parenting practices, all but one of these (permissive discipline) were marked by significant differences between the different emotional intelligence profiles. In the case of the mother’s parenting practices, only three of these, all of them positive, had significantly different scores with respect to the different emotional intelligence profiles.

In this line of results, recent studies (García-Linares et al. 2011; Nishikawa et al. 2010) have asserted that the father’s behavior toward the adolescent is as important to the child’s well-being as the mother’s. In addition, the idea that the importance of the father’s attachment increases with age (Williams and Kelly 2005) seems to be confirmed in this study.

The limitations of the present study, relating to sample representativeness and the fact that the college students were assessed using self-report measures, could be improved upon, thus suggesting prospects for future research work. Additionally, instead of obtaining information from a single source, another improvement would be to use other informers, both for parenting practices (e.g., the parents themselves) and for emotional intelligence. It would also be beneficial to carry out longitudinal studies to determine the developmental course of emotional intelligence.

The results of this study, although only a first approximation, and limited by the effect size found, can nonetheless offer relevant information towards the treatment and prevention of emotional problems in early adulthood. Parents should become aware of the importance of their parenting practices in the development of their children’s emotional behavior, and their influence in the development of dysfunctional patterns that may affect their children’s psychological well-being.

Finally, it would be advisable for future studies to explore why the role of the father is more relevant than that of the mother in the emotional intelligence of the children. In addition, it would be useful to check whether the result of this research is also obtained in other cultures, since the significance of educational practices may be different depending on cultural factors.
